# Adverse childhood experiences and pre-pregnancy body mass index in the HUNT study: A population-based cohort study

**DOI:** 10.1371/journal.pone.0285160

**Published:** 2023-05-02

**Authors:** Heidi Linn Sandsæter, Trine Tetlie Eik-Nes, Linn Okkenhaug Getz, Elisabeth Balstad Magnussen, Ottar Bjerkeset, Janet W. Rich-Edwards, Julie Horn

**Affiliations:** 1 Department of Public Health and Nursing, Norwegian University of Science and Technology, Trondheim, Norway; 2 Department of Obstetrics and Gynecology, Levanger Hospital, Nord-Trøndelag Hospital Trust, Levanger, Norway; 3 Department of Neuromedicine and Movement Science, Norwegian University of Science and Technology, Trondheim, Norway; 4 Stjørdal Community Mental Health Centre, Levanger Hospital, Levanger, Norway; 5 Department of Public Health and Nursing, General Practice Research Unit, Norwegian University of Science and Technology, Trondheim, Norway; 6 Department of Obstetrics and Gynecology, St. Olav’s University Hospital, Trondheim, Norway; 7 Department of Clinical and Molecular Medicine, Norwegian University of Science and Technology, Trondheim, Norway; 8 Faculty of Nursing and Health Sciences, Nord University, Levanger, Norway; 9 Department of Mental Health, Norwegian University of Science and Technology, Trondheim, Norway; 10 Department of Epidemiology, Harvard T.H. Chan School of Public Health, Boston, MA, United States of America; 11 Department of Medicine, Brigham and Women’s Hospital, Division of Women’s Health and Connors Center for Women’s Health and Gender Biology, Boston, MA, United States of America; Tulane University School of Public Health and Tropical Medicine, UNITED STATES

## Abstract

**Objective:**

Investigate the association between adverse childhood experiences and pre-pregnancy body mass index (BMI) in a population-based cohort in Trøndelag county, Norway.

**Materials and methods:**

We linked data from the third (2006–2008) or fourth (2017–2019) survey of the Trøndelag Health Study (HUNT) and the Medical Birth Registry of Norway for 6679 women. Multiple logistic regression models were used to examine the association between adverse childhood experiences and pre-pregnancy BMI. Adverse childhood experiences were self-reported in adulthood and included perceiving childhood as difficult, parental divorce, parental death, dysfunctional family environment, bad childhood memories and lack of support from a trusted adult. Pre-pregnancy BMI was derived from the Medical Birth Registry of Norway or BMI measurement from the HUNT survey conducted within 2 years prior to the woman’s pregnancy.

**Results:**

Perceiving childhood as difficult was associated with higher odds of pre-pregnancy underweight (OR 1.78, 95%CI 0.99–3.22) and obesity (OR 1.58, 95%CI 1.14–2.2). A difficult childhood was positively associated with obesity with an adjusted OR of 1.19, 95%CI 0.79–1.81 (class I obesity), 2.32, 95%CI 1.35–4.01 (class II obesity) and 4.62, 95%CI 2.0–10.65 (class III obesity). Parental divorce was positively associated obesity (OR 1.34, 95%CI 1.10–1.63). Bad childhood memories were associated with both overweight (OR 1.34, 95%CI 1.01–1.79) and obesity (OR 1.63, 95%CI 1.13–2.34). Parental death was not associated with pre-pregnancy BMI.

**Conclusions:**

Childhood adversities were associated with pre-pregnancy BMI. Our results suggest that the positive associations between childhood adversities and pre-pregnancy obesity increased with increasing obesity level.

## Introduction

Obesity is among the world’s greatest health challenges, including among women of reproductive age [1]. Worldwide, the prevalence of obesity in women has increased from seven percent in 2000 to fifteen percent in 2016 [2]. This trend also affects pre-pregnancy body mass index (BMI) development [1]. Obesity is now one of the most common risk factors in obstetric practice [1], and is associated with adverse maternal and neonatal outcomes both in short and long term [3,4]. Increased screening and monitoring of pregnancy and childbirth in women with obesity is therefore recommended [5], entailing extra examinations, interventions and involvement of many health professionals. Hence, a risk-focused prevention in antenatal care may inadvertently expose women with obesity to potentially stressful and alienating follow-up and procedures [6].

The causal pathways to obesity are highly complex [7]. There is however a widespread, simplistic belief that obesity can be explained by high calorie intake and low physical activity alone [8,9]. Previous studies have found that a history of adverse childhood experiences (ACEs) is associated with adult obesity [10,11], but the role of ACEs on the life course development of body weight is incompletely understood. Transition to parenthood is a strong determinant of life course weight trajectories [12]. Evaluating the relationship between ACEs and pre-pregnancy BMI can therefore provide a better understanding of how ACEs affect weight development over the life course and how this might influence antenatal care.

ACEs typically involve physical-, sexual- and emotional abuse, physical and emotional neglect, and dysfunctional household [13]. Further, ACEs have been linked to obesity through different mechanisms including allostatic overload, which demonstrates how toxic stress may overstrain individual’s physiological adaptation mechanisms [14,15]. Traumatic experiences during pregnancy may increase women’s vulnerability for reactivation of ACEs [16].

Previous studies investigating the relationship between ACEs and pre-pregnancy BMI have mostly been limited by relatively small sample sizes and clinical-based samples, mainly focusing on the association between ACEs, overweight and obesity [17–21]. We therefore aim to investigate the association between childhood quality and pre-pregnancy BMI, including underweight, in a large population-based sample in Norway.

## Method

We conducted a retrospective study, linking data from the population based Trøndelag Health Study (the HUNT Study) with information from the Medical Birth Registry of Norway (MBRN).

### Study population

The HUNT Study is an ongoing population-based cohort study in the Northern part of Trøndelag county in Norway. All residents aged 20 years or older have been invited to four health surveys: HUNT1 (1984–86), HUNT2 (1995–97), HUNT3 (2006–08), and HUNT4 (2017–19) [22]. All participants in HUNT completed self-administrated questionnaires on aspects of physical and mental health, socio-economic factors and family history, and had a clinical examination that included standardized measurements of height and weight. The questionnaires used in HUNT3 and HUNT4 included questions on childhood quality.

The MBRN is a compulsory registry containing detailed information on all live and still-birth after 16 weeks of gestation since 1967. The registration is based on a standardized form completed by midwives or doctors at the delivery units [23]. Information on women’s height and pre-pregnancy weight have been systematically recorded in the MBRN since 2007. The recording was very scarce from 2007 and the following years. In 2012, the MBRN had information on pre-pregnancy weight for only 18% of pregnant women in Trøndelag county. The proportion increased to 60% in 2017, and reached 90% in 2021 [24].

**Fig 1:**
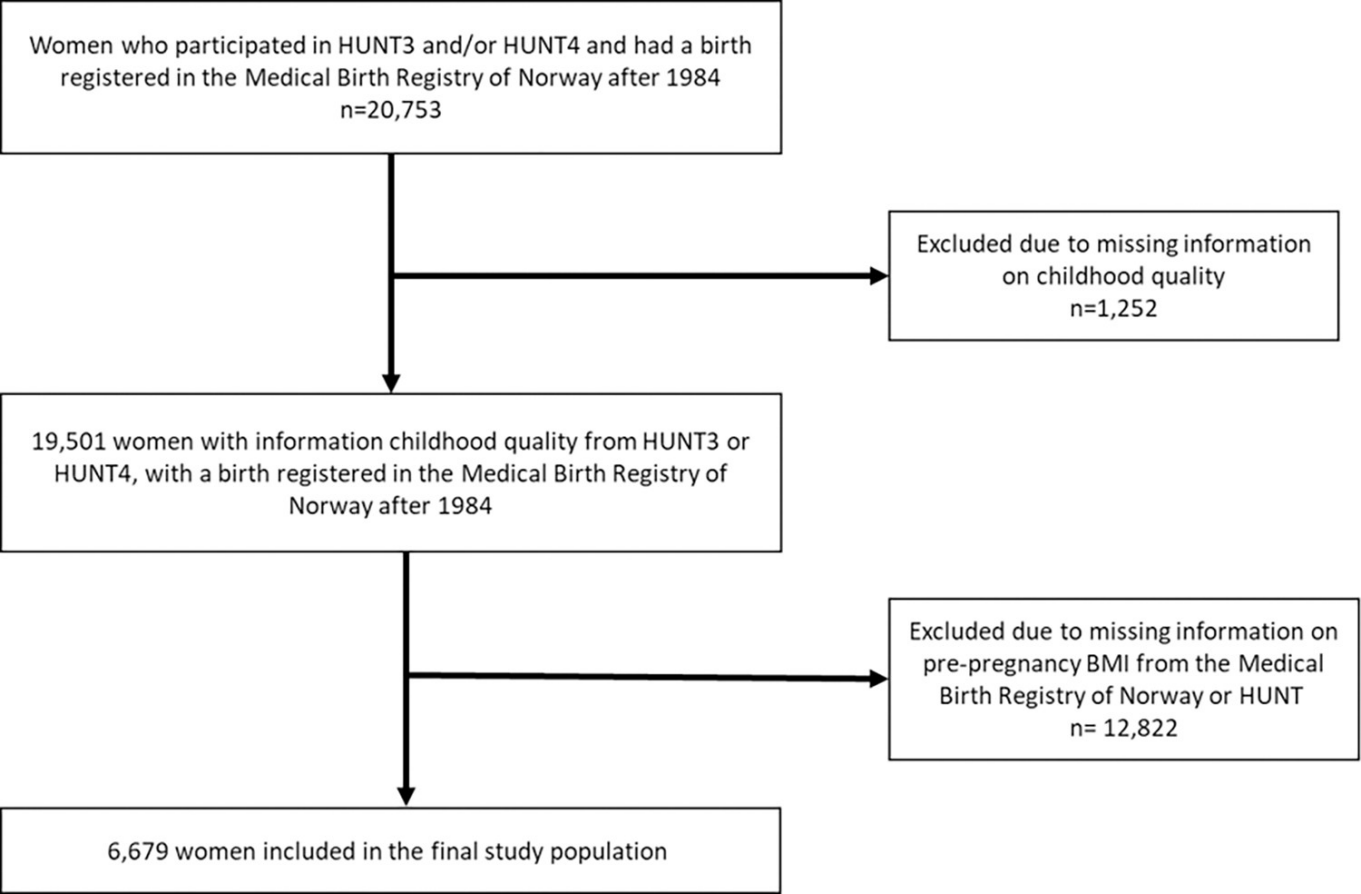
Flow chart of the study population.

Our study population included 20,753 female participants from HUNT3 and HUNT4 with one or more deliveries recorded in the MBRN between 1984 (start of HUNT1) and December 31^st^, 2019. Every participant contributed once to the analysis with information from the first delivery with available data on pre-pregnancy BMI. We excluded 1,252 women due to missing information on childhood quality and 12,822 women with missing information on pre-pregnancy BMI. After these exclusions, 6,679 women were eligible for inclusion (Fig 1). Descriptive characteristics of excluded versus included women are shown in S1 Table.

### Measures

We used a childhood quality questionnaire from HUNT3 and HUNT4, based on the Difficult Childhood Questionnaire (DCQ) developed for HUNT3 [25,26]. A Norwegian study including 28,047 individuals confirmed that the questionnaire used in this study is an empirically supported method of assessment of ACEs [26].

The childhood quality questionnaire included the following six questions:

1. When you think about your childhood, would you describe it as: Very good–good–moderate–difficult–very difficult?
2. Parental divorce during childhood or adolescent?
3. Parental death during childhood or adolescents?
4. Did you experience a lot of arguing, turmoil, conflicts, or difficult communication in your childhood home?
5. Do you struggle with bad memories from your childhood, due to loss, betrayal, neglect, violence, ill-treatment, or abuse?
6. Growing up, did you have a trusted adult from whom you could get support?

Questions 1–3 were asked in both HUNT3 and HUNT4. In HUNT4 questions 4–6 were included to further assess the childhood environment.

Information on self-reported quality of childhood (item 1), dysfunctional family environment (item 4), bad memories in childhood (item 5) and lack of support from a trusted adult (item 6) were reported on a 5-point Likert scale ranging from “very good” to “very difficult” and from “to a very high degree” to “not at all”. Parental divorce and/or death during childhood were reported as: No-yes, before I was 7-yes, when I was 7–18 years old. To avoid small samples in the subgroups, information on the different exposures was dichotomized into: “very good/good/moderate” or “difficult/very difficult” and “to a very high degree/to a large extent” or “to a small degree/to a very small extent/not at all”. Parental divorce or death was dichotomized as “yes or no”. For women with available data from both HUNT3 and HUNT4, we used information from HUNT3.

We included data on pre-pregnancy body mass index (BMI) from the MBRN and HUNT. For women with available data from both sources, we prioritized BMI from the MBRN to capture woman’s BMI closer to the beginning of pregnancy. Pre-pregnancy BMI was calculated as weight divided by the squared value of height (kg/m^2^) and categorized into underweight (< 18.5 kg/m^2^), normal weight (18.5 to < 25 kg/m^2^), overweight (25.0 to < 30 kg/m^2^) or obesity (≥ 30 kg/m^2^). We further subdivided obesity into obesity class l (30- <35 kg/m^2^), obesity class ll (35- <40 kg/m^2^) and obesity class lll (≥ 40 kg/m^2^).

For 3,844 (57.5%) women lacking information on pre-pregnancy BMI from the MBRN, we used the first available BMI measurement from the HUNT survey conducted within two years prior to the woman’s pregnancy. Trained staff at the HUNT field stations performed standardized weight and height measurements of the participants wearing light clothes without shoes. For some participants BMI data from a Young-HUNT survey within two years before pregnancy were used. All adolescents attending junior or senior high school (13–19 years) were invited to participate in the adolescent part of the HUNT Study: Young-HUNT1 (1995–97), Young-HUNT2 (1999–00), Young-HUNT3 (2006–08), and Young-HUNT4 (2017–19) [27]. The surveys were completed during class hours.

### Statistical analysis

The association between exposure to ACEs and pre-pregnancy BMI category (the outcome of interest) was assessed using multiple logistic regression. Separate logistic regression analyses were conducted for each outcome (underweight, overweight, obesity, obesity class I-III) with the normal weight group as the reference group. Covariates included in the models were selected based on prior knowledge and directed acyclic graphs (S1 Fig). Analyses were adjusted for maternal age and maternal birthyear which were included as continuous covariates.

To assess misclassification of exposure, we performed a sensitivity analysis restricted to participants born >1980, as older women might be more likely to under-report the occurrence of ACEs [10]. More, we examined the potential impact of inconsistencies in the reporting of childhood quality among women with repeated information on ACEs (HUNT3 and HUNT4). In this analysis, we contrasted the associations between women’s first reported ACEs (HUNT3) versus their worst reported ACEs (HUNT3 or HUNT4) and prepregnancy BMI. Information on maternal prepregnancy BMI in the MBRN is based on self-reported pre-pregnancy weight or non-standardized weight measurements in the first trimester. To address the potential of misclassification of pre-pregnancy BMI in the MBRN, we evaluated concordance between pre-pregnancy BMI values reported to the MBRN (BMI_MBRN_) and pre-pregnancy BMI measured at HUNT (BMI_HUNT_) for 326 participants with available data from both sources using Intraclass correlation coefficient (ICC). Mean differences between BMI_MBRN_ and BMI_HUNT_ were reported by BMI category. We also assessed the proportion agreement between BMI categories according to BMI_MBRN_ and BMI_HUNT_. All analyses were performed using Stata, version 17 (StataCorp, College Station, TX USA).

### Ethics

All participants in the HUNT study provided written informed consent. The present study was approved by the Regional Committee for Ethics in Medical Research (REC Central, reference number: 18.10.19/30294).

## Results

In our sample of 6,679 study participants, 4,223 (63.2%) women were normal weight before pregnancy, 154 (2.3%) underweight, 1,577 (23.6%) overweight, and 725 (10.9%) categorized with obesity (Table 1).

**Table 1:** Descriptive characteristics of the study population (n = 6,679).

		Pre-pregnancy BMI				
Maternal characteristics*		Underweight (n = 154)	Normal weight (n = 4,223)	Overweight (n = 1,577)		Obesity (n = 725)
Birthyear, median (IQR)		1973 (1961–1987)	1974 (1961–1985)	1978 (1968–1986)		1981 (1972–1987)
Maternal age, mean (SD)		27.2 (4.6)	29.1 (4.6)	29.6 (4.8)		30.2 (5.1)
Parity, n (%)						
	Nulliparous	72 (46.8)	1,621 (38.4)	600 (38.1)		268 (37.0)
	Para 1	53 (34.4)	1,532 (36.3)	562 (35.6)		254 (35.0)
	Para 2+	29 (18.8)	1,070 (25.3)	415 (26.3)		203 (28.0)
Marital status, n (%)						
	Married, cohabitant	139 (90.3)	3,936 (93.2)	1,486 (94.2)		689 (95.0)
	Divorced, widowed, separated	0	22 (0.5)	4 (0.3)		2 (0.3)
	Single	15 (9.7)	255 (6.0)	84 (5.3)		32 (4.4)
	Missing	0	10 (0.2)	3 (0.2)		2 (0.3)
Education, n (%)						
	Lower secondary (≤9 years)	5 (3.3)	151 (3.6)	45 (2.9)		45 (6.2)
	Upper secondary (10–12 years)	88 (57.1)	1,684 (39.9)	664 (42.1)		319 (44.0)
	Tertiary (>12 years)	57 (37.0)	2,291 (54.3)	838 (53.1)		348 (48.0)
	Missing	4 (2.6)	97 (2.3)	30 (1.9)		13 (1.8)

* Information on birthyear, maternal age, parity, marital status and employment status from The Medical Birth Registry of Norway (MBRN). Information on education from HUNT surveys.

**Fig 2:**
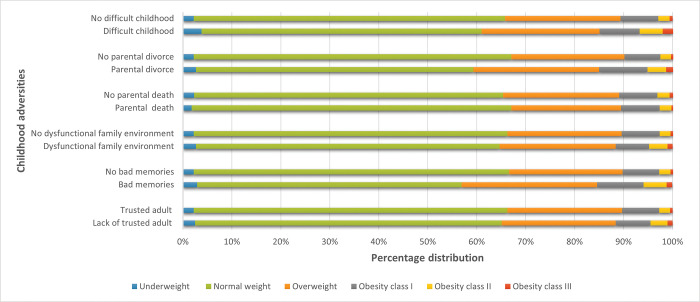
Pre-pregnancy BMI according to childhood adversities.

Compared with women with normal pre-pregnancy BMI, women who were underweight before pregnancy were slightly younger, reported lower education levels and were more likely to be primiparous or unmarried. Women with pre-pregnancy obesity were older, reported lower education levels and were more likely to be married compared with women who were normal weight before pregnancy (Table 1). Fig 2 displays the distribution of pre-pregnancy BMI according to childhood adversities. Except for parental deaths, the prevalence of pre-pregnancy obesity was higher among women who reported ACEs compared to those who reported no ACEs.

The associations between ACEs and pre-pregnancy BMI are shown in Table 2 (adjusted ORs) and S2 Table (crude ORs). In the fully adjusted models, including adjustment for maternal age and birthyear, a difficult childhood was associated with higher odds of pre-pregnancy underweight (OR 1.78, 95%CI 0.99–3.22) and obesity (OR 1.58, 95%CI 1.14–2.20) compared to those with normal weight pre-pregnancy. A difficult childhood was positively associated with obesity and when obesity was stratified by severity estimates increased with increasing obesity level, with an adjusted OR of respectively 1.19, 95%CI 0.79–1.81 (class I obesity), 2.32, 95%CI 1.35–4.01 (class II obesity) and 4.62, 95%CI 2.0–10.65 (class III obesity). Likewise, parental divorce during childhood was associated with a greater odds of obesity (OR 1.34, 95%CI 1.10–1.63). Bad childhood memories were associated with both overweight (OR 1.34, 95%CI 1.01–1.79) and obesity (OR 1.63, 95%CI 1.13–2.34). In analyses stratified by obesity level, dysfunctional family environment and lack of trusted adult were both associated with higher odds for obesity class II (OR 1.62, 95%CI 1.05–2.47) and (OR 1.68, 95%CI 1.10–2.58), respectively.

**Table 2:** Odds ratios and 95% confidence intervals of pre-pregnancy BMI according to adverse childhood experiences.

Adverse childhood experiences			n (total)	n (exposed)	OR*	95% CI
**Perceiving childhood as difficult**			6,679	341		
	Normal weight (BMI 18.5–24.9)		4,223	195	1.00	-
	Underweight (BMI <18.5)		154	13	1.78	0.99–3.22
	Overweight (BMI 25–29.9)		1,577	82	1.15	0.88–1.50
	Obese (BMI ≥30)		725	51	1.58	1.14–2.20
		Obesity class 1 (BMI 30–34.9)	518	28	1.19	0.79–1.81
		Obesity class 2 (BMI 35–39.9)	164	16	2.32	1.35–4.01
		Obesity class 3 (BMI ≥40)	43	7	4.62	2.0–10.65
**Parental divorce during childhood**			6,661	1,251		
	Normal weight (BMI 18.5–24.9)		4,214	708	1.00	-
	Underweight (BMI <18.5)		153	34	1.32	0.87–1.99
	Overweight (BMI 25–29.9)		1,569	321	1.11	0.95–1.30
	Obese (BMI ≥30)		723	188	1.34	1.10–1.63
		Obesity class 1 (BMI 30–34.9)	517	124	1.21	0.97–1.52
		Obesity class 2 (BMI 35–39.9)	164	47	1.48	1.03–2.13
		Obesity class 3 (BMI ≥40)	42	17	2.90	1.51–5.57
**Parental death during childhood**			6,664	331		
	Normal weight ((BMI 18.5–24.9)		4,212	216	1,00	-
	Underweight (BMI <18.5)		154	6	0.75	0.33–1.73
	Overweight (BMI 25–29.9)		1,574	74	0.97	0.74–1.28
	Obese (BMI ≥30)		724	35	1.06	0.73–1.55
		Obesity class 1 (BMI 30–34.9)	517	26	1.11	0.72–1.70
		Obesity class 2 (BMI 35–39.9)	164	8	1.09	0.53–2.27
		Obesity class 3 (BMI ≥40)	43	1	0.49	0.07–3.63
**Dysfunctional family environment**			5,575	768		
	Normal weight ((BMI 18.5–24.9)		3,557	476	1.00	-
	Underweight (BMI <18.5)		125	21	1.26	0.78–2.05
	Overweight (BMI 25–29.9)		1.302	182	1.00	0.83–1.21
	Obese (BMI ≥30)		591	89	1.04	0.81–1.34
		Obesity class 1 (BMI 30–34.9)	425	52	0.82	0.60–1.11
		Obesity class 2 (BMI 35–39.9)	135	29	1.62	1.05–2.47
		Obesity class 3 (BMI ≥40)	31	8	2.01	0.89–4.56
**Struggle with bad memories**			5,571	274		
	Normal weight ((BMI 18.5–24.9)		3,558	148	1.00	-
	Underweight (BMI <18.5)		125	8	1.46	0.69–3.05
	Overweight (BMI 25–29.9)		1,302	76	1.34	1.01–1.79
	Obese (BMI ≥30)		586	42	1.63	1.13–2.34
		Obesity class 1 (BMI 30–34.9)	421	26	1.38	0.89–2.14
		Obesity class 2 (BMI 35–39.9)	135	13	2.23	1.22–4.09
		Obesity class 3 (BMI ≥40)	30	3	2.60	0.77–8.79
**Lack of trusted adult during childhood**			5,552	840		
	Normal weight ((BMI 18.5–24.9)		3,544	526	1.00	-
	Underweight (BMI <18.5)		124	21	1.15	0.71–1.86
	Overweight (BMI 25–29.9)		1.300	196	1.05	0.88–1.26
	Obese (BMI ≥30)		584	97	1.18	0.93–1.51
		Obesity class 1 (BMI 30–34.9)	420	60	0.99	0.74–1.33
		Obesity class 2 (BMI 35–39.9)	134	29	1.68	1.10–2.58
		Obesity class 3 (BMI ≥40)	30	8	2.05	0.90–4.69

BMI, body mass index; CI, confidence interval; OR, odds ratio.

*Models are adjusted for age and birthyear.

When restricting our analyses to women born after 1980, associations between ACEs and pre-pregnancy BMI appeared to be slightly stronger (S3 Table). For women with repeated information on childhood quality from HUNT3 and HUNT4, we observed similar estimates for associations between first reported and worst reported ACEs with pre-pregnancy BMI, implying that inconsistencies in the reporting of childhood quality had not influenced the results (S4 Table). Our analyses showed strong correlation (ICC = 0.96) between BMI_MBRN_ and BMI_HUNT_. Despite a slight tendency towards MBRN overestimating BMI in women with underweight and underestimating BMI in women with overweight and obesity (S2 Fig), most women were classified in the same BMI category when BMI_MBRN_ was used instead of BMI_HUNT_ (S5 Table).

## Discussion

In this population-based cohort, we observed higher odds of both pre-pregnancy underweight and pre-pregnancy obesity among women with a difficult childhood. Similarly, parental divorce and struggling with bad memories was associated with higher odds of pre-pregnancy obesity. For all ACEs except parental death during childhood, odds were highest for pre-pregnancy class lll obesity, suggesting a dose-response relationship.

Overall, our results are consistent with previous studies of associations between ACEs and pre-pregnancy BMI [17–21]. In a large U.S. sample including 2,873 mothers with retrospective information of ACEs, Ranchod et al. found 60% higher risk of pre-pregnancy obesity among women who reported childhood physical abuse [20]. Likewise, a cross-sectional study in Germany including 326 women, reported an association between physical abuse and higher odds of pre-pregnancy overweight and obesity [17]. Hollingsworth et al. examined the associations between emotional or physical abuse and risk of pre-pregnancy weight in a cross sectional study of 239 Australian women, which found an associated with pre-pregnancy obesity but not with pre-pregnancy overweight [19]. This is in line with our results of higher odds of pre-pregnancy obesity than pre-pregnancy overweight. While previous studies have been unable to distinguish between different classes of pre-pregnancy obesity, we found increasing odds for more severe obesity in pre-pregnancy, consistent with findings from a large study in US adults which reported strongest associations between ACEs and obesity in women with class III obesity [28].

In contrast to previous studies with smaller sample sizes, we were able to examine the associations between ACEs and pre-pregnancy underweight. Although not statistically significant owing to small subgroups with underweight, we found that all ACEs, except parental death, appeared to be associated with higher risk of pre-pregnancy underweight. While there are no previous studies evaluating the association of ACEs and pre-pregnancy underweight, there are several studies examining the relationship between ACEs and underweight in children and adolescents providing conflicting results. For example, a study investigating association between ACEs and weight status among more than 100, 000 adolescents from the Minnesota Student Survey, found no association between ACEs and underweight [28]. In contrast, a recent Polish cohort study reported a greater risk of both underweight and obesity in a general population of children with experience of ACEs [29]. Similarly, a meta-analysis suggested a greater risk of anorexia in women who experienced physical abuse in childhood [30].

Our findings showing no association between parental death and pre-pregnancy BMI are consistent with a large cohort study in Danish children. Danish children bereaved by the loss of a parent through the first six years of life had similar BMI and similar risk of overweight at 7–13 years compared to those who did not experience this loss [31]. A possible explanation can be that parental death doesn’t represent humiliation and unwanted attention directed to the child itself. Further, death represents a more distinct event, limited to a shorter period, often triggering massive support from the environment.

In contrast to parental death, parental divorce during childhood and adolescence was associated with both pre-pregnancy underweight and obesity, with increasing odds in the more severe obesity classes. Our findings are in accordance with two previous studies from the UK and Denmark, which reported higher BMI and increased risk of overweight and obesity in children who had experienced parental separation [32,33]. Parental divorce may be a difficult and long-lasting process rather than a distinct incident, thus affecting children’s lives negatively for years after parental separation, possibly related to economic decline, post-separation conflicts and loss of parental support, giving rise to a feeling of worthlessness and negative effects on mental health [26,34,35].

Previous studies from the US, Germany and Australia examining the relationship between childhood adversities and pre-pregnancy BMI, up to 53 (between 13 and 53) % of the participants reported a history of ACEs [17–21]. In contrast, only 4.4% of the women in our sample experienced their childhood as difficult, suggesting a less widespread exposure to ACEs in the HUNT population. This may be explained by differences in study populations and assessment of ACEs.

The Difficult Childhood Questionnaire used in our study provided information of women´s overall assessment of their childhood quality, rather than specific abuse or neglect experiences. Asking for women’s subjective perception of childhood quality might reflect an overall balance between resilience factors and damaging factors, perceived as toxic stress known to affect childhood quality negatively.

Previous studies on the relationship between ACEs and pre-pregnancy BMI have examined the distinct influence of several types of abuse or neglect, number of different experiences, and stages of severity on pre-pregnancy BMI [36,37]. In this study, women´s assessment of their childhood quality or difficult memories may include various ACEs and also possibly an interaction between the adverse experiences. Previous studies have shown that ACEs tend to cluster [36,37], and the presence of one ACE increases the risk of exposure to additional ACEs [37]. Furthermore, many important adversities are still not represented in the different questionnaires assessing ACEs [38,39]. However, results from our study suggest that a subjective, global evaluation of the childhood in a non-intrusive questionnaire can provide clinically relevant information for the association between ACEs and pre-pregnancy BMI.

Our sample consisted of a relatively homogenous population in Norway, while many previous studies included women from prenatal clinics with higher rates of low-income or mental health problems [19,21]. Using the same childhood quality questionnaire as in our study, Haugland et al. described that 10% of the women in a general population in Norway characterized their childhood as difficult [26]. However, unlike our study, Haugland et al. conducted their study in an area characterized by higher rates of unemployment and young people seeking help for mental health problems.

Theories suggest that exposure to chronic stress may lead to “allostatic overload” if individuals are exposed to chronic stress without the possibility to recover [14]. The theory of allostatic overload shed light on how an imbalance in “the wear and tear on the body” can promote obesity through chronic activation of the stress response system, causing autonomic neuroendocrine and inflammatory dysfunction [15].

Further, a model of socioeconomic disadvantage where adult distress can lead to a disharmonious family environment, may explain a possible psycho-emotional overload in children [40]. The consequences may subsequently be disturbance of physiological homeostasis leading to disruption in energy balance, with elevated appetite, reduced energy expenditure and disrupted eating pattern and escalation of weight [40,41]. A strong association between ACEs and disturbed eating pattern embodied as food addiction was shown in a large sample in the Nurses’ Health Study Il [42]. In the same sample they found posttraumatic stress disorder (PTSD) to be a strong predictor of food addiction especially when PTSD symptoms occurred in early childhood [43].

Furthermore, a large population-based, cross-sectional survey in US children aged six to seventeen showed negative associations between ACEs and five healthy weight behaviors. The odds of involving in a healthy behavior decreased in a dose-response manner when exposed to more ACEs [44]. These results supports that children exposed to ACEs with disturbed stress responses impacting the hypothalamic–pituitary–adrenal (HPA) axis [45] grow up with decreased opportunities to adopt a healthy lifestyle [44].

### Strengths and limitations

This is one of the first studies investigating the associations between ACEs and pre-pregnancy BMI in a large population-based cohort. Further strengths include the linkage to a national birth registry and availability of measured, rather than self-reported, data on pre-pregnancy weight. Previous studies examining the association of ACEs and pre-pregnancy BMI have usually differentiated between single types of adversities. In this study however, a brief ACE measure based on non-intrusive items emphasizes women’s subjective assessment of their childhood quality.

For all ACEs except for parental death our estimates suggested sound statistical evidence of associations with increased risk of pre-pregnancy underweight. However, these results should be interpreted with caution since they were hampered by low numbers and did not reach statistical significance at the chosen level (95% CI). A limitation of this study is the retrospective recall and results should thus be interpreted with caution as we do not know the exact timing of the ACE exposure and weight trajectory. Furthermore, the retrospective assessment of ACEs is prone to recall bias. However, a review paper examining the validity of retrospective recall of abuse, neglect, and family discord suggests that retrospective reports should lead to downwardly biased estimates [10]. Like Haugland et al. we found the highest prevalence of ACEs in women in their thirties, while the prevalence decreased by age [26]. Especially older participants might have underreported ACEs, which is consistent with our observation of stronger associations among younger study participants. We used data on pre-pregnancy BMI from two different sources. However, comparison of pre-pregnancy BMI measured at HUNT participation with pre-pregnancy BMI reported to the MBRN showed a high correlation (0.96). As in all observational studies, we cannot rule out the possibility of residual confounding including confounding by childhood socioeconomic status (SES), which we were unable to control for due to large amount of missing information on family income in childhood. Furthermore, we were not able to adjust for other early life factors such as birthweight, medical family history or family lifestyle. Unfortunately, this information is lacking in most prior studies in this field [17–19,21]. A large meta-analysis reported that adjustment for childhood SES made little difference in the association between ACEs and obesity [11]. Since women`s education and employment are considered as mediators, and because we aimed to capture the total association between ACEs and pre-pregnancy weight, we did not adjust for these covariates. Furthermore, our analysis is not adjusted for parity since we considered this to be a potential collider rather than a confounder.

The HUNT population is considered as fairly representative for the Norwegian population [27]. Since the HUNT population is ethnically homogeneous and few participants in this sample have experienced severe social deprivation or racial or ethnic discrimination, the generalizability of our findings may be limited with respect to more diverse populations.

## Conclusion

In this large, population-based sample in Norway, we observed an j-shaped association between childhood adversities and pre-pregnancy BMI.

Based on this knowledge, healthcare providers in maternity care should be sensitive to ACEs among pregnant women with both underweight and obesity. Individualized, trauma-sensitive care may prevent weight bias and promote trust and safety inducing consultations. Recommendations about weight management and follow-up for these women might benefit from an increased awareness of the complexity of weight development, including ACEs and facilitate better reproductive health and promote health across generations [46].

Further research should aim for a better understanding of the critical periods for excessive weight gain or weight loss in women with ACEs. In addition, more knowledge on how co-occurrence of different ACEs affects weight development in girls and women in reproductive age is needed. In-depth exploration of these women’s perspectives of weight development throughout their life courses and their experience of pregnancy care, can provide the research field with new knowledge from women’s’ experiences when translating results into practical maternity care guidelines.

## Supporting information

S1 Fig Directed acyclic graph of the association between Adverse childhood experiences and pre-pregnancy BMI. (TIF)

S2 Fig Box plots of the difference between BMI_MBRN_ and BMI_HUNT_. (TIF)

S1 Table Descriptive characteristics of female HUNT participants who had a birth registered in the MBRN between 1984–2019 by inclusion status. (DOCX)

S2 Table Crude odds ratios and 95% confidence intervals of pre-pregnancy BMI according to adverse childhood experiences. (DOCX)

S3 Table Associations of maternal childhood experiences with pre-pregnancy BMI in a subpopulation of younger women born after 1980. (DOCX)

S4 Table Associations of perceiving childhood as difficult with pre-pregnancy BMI in a subpopulation of women with repeated information from HUNT3 and HUNT4. (DOCX)

S5 Table Categorization of pre-pregnancy BMI category based on BMI reported to the MBRN and BMI measured at HUNT (n = 326). (DOCX)
