# Supplementary figures and images for: Adverse childhood experiences and pre-pregnancy body mass index in the HUNT study: A population-based cohort study
Source: PLoS One. 2023 May 2;18(5):e0285160. doi: 10.1371/journal.pone.0285160 (PMC10153725; doi:10.1371/journal.pone.0285160)

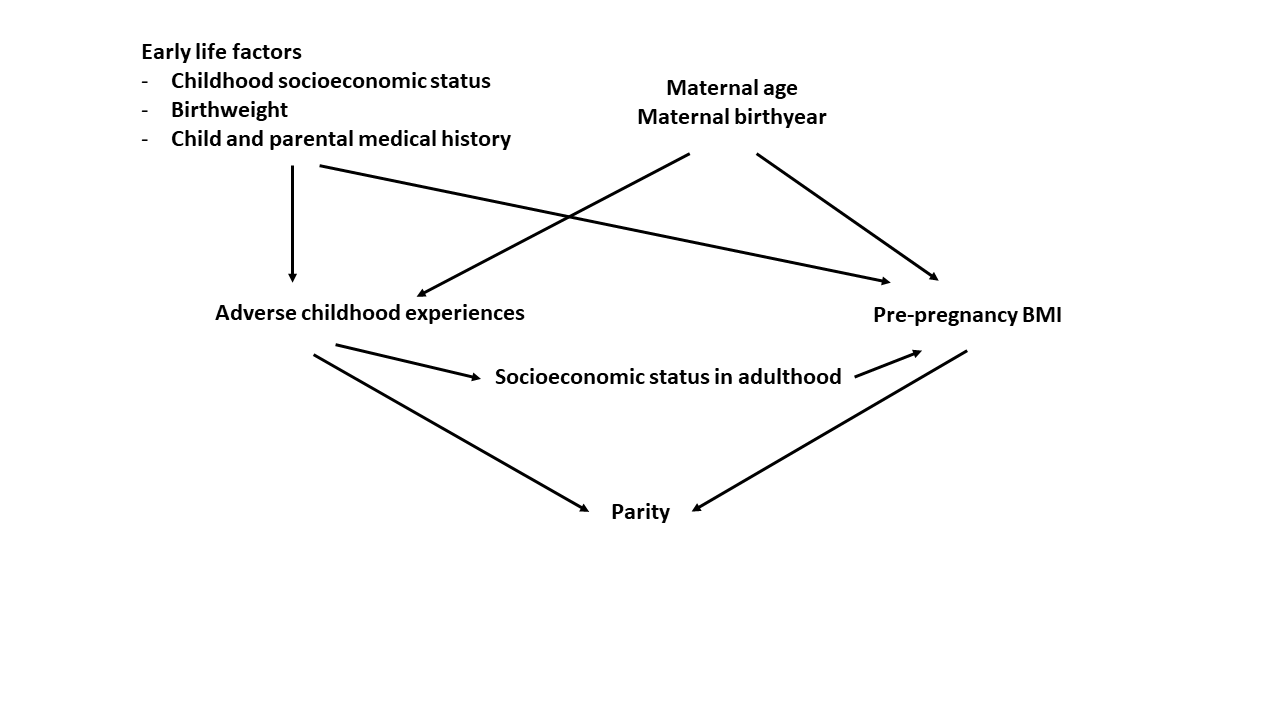

Supplement: S1 Fig — (TIF) [file pone.0285160.s001.tif]

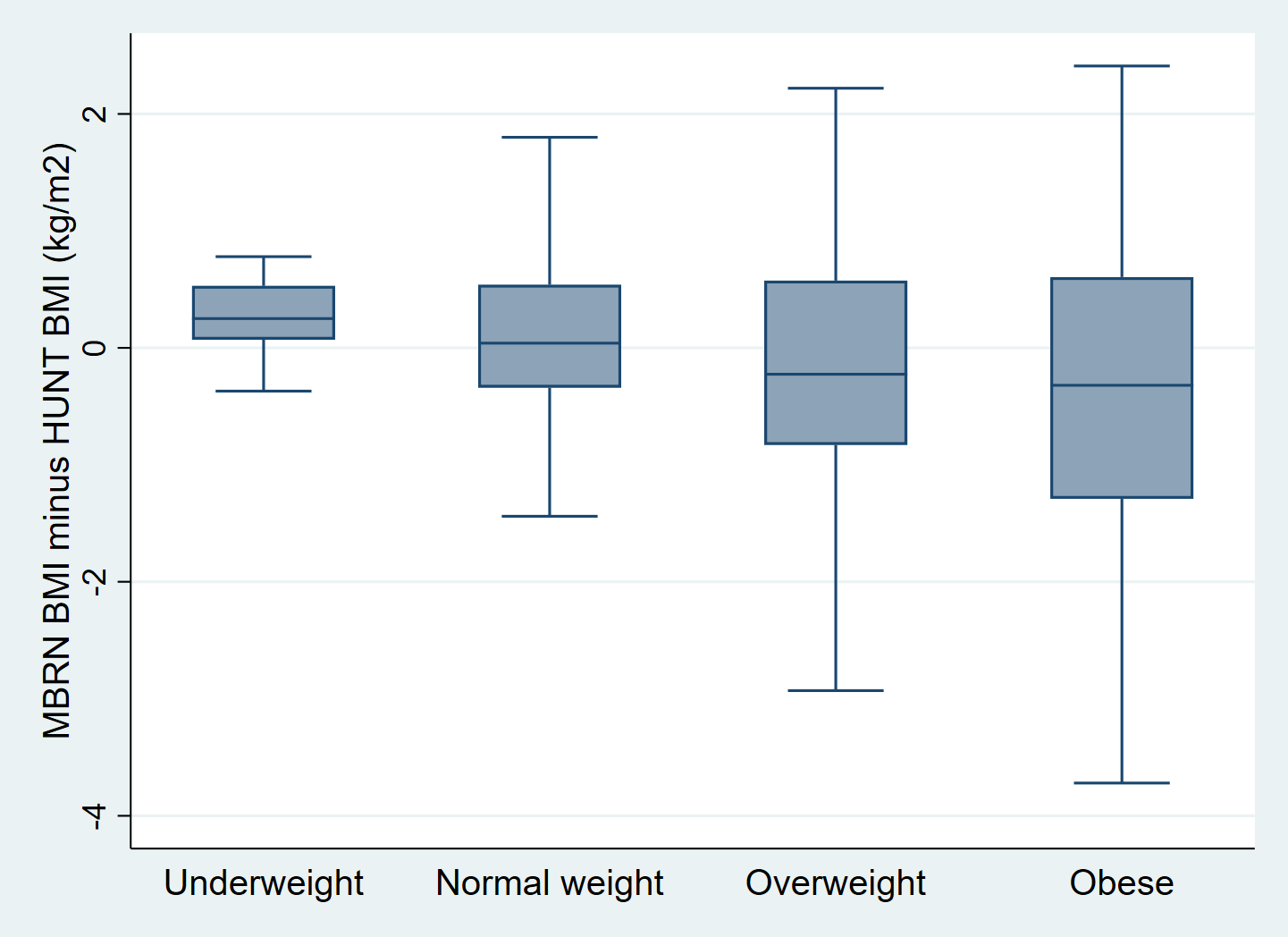

Supplement: S2 Fig — (TIF) [file pone.0285160.s002.tif]
