# Supplementary material for: Adverse childhood experiences and pre-pregnancy body mass index in the HUNT study: A population-based cohort study
Source: PLoS One. 2023 May 2;18(5):e0285160. doi: 10.1371/journal.pone.0285160 (PMC10153725; doi:10.1371/journal.pone.0285160)
Supplement: S1 Table — (DOCX) [file pone.0285160.s003.docx]

| **S1 Table. Descriptive characteristics of female HUNT participants who had a birth registered in the MBRN between 1984-2019 by inclusion status.** | | | | | |
| --- | --- | --- | --- | --- | --- |
|  | | Included in the analyses  (n=6,679) |  | Excluded from the analyses  (n=14,074) | ***p-value |
| Maternal characteristics** | |  | | |  |
| Birthyear, median (SD) | | 1970 (11.2) |  | 1974 (12.5) | <0.001 |
| Maternal age, mean (SD) | | 31,1 (4.5) |  | 30,1 (4.9) | <0.001 |
| Parity, n (%) | |  |  |  | <0.001 |
|  | Nulliparous | 934 (14.0) |  | 2,454 (17.4) |  |
|  | Para 1 | 2,704 (40.5) |  | 6,250 (44.4) |  |
|  | Para 2+ | 3,041 (45.5) |  | 5,370 (38.2) |  |
| Marital status, n (%) | |  |  |  | <0.001 |
|  | Married, cohabitant | 6,412 (96.3) |  | 13,159 (93.5) |  |
|  | Divorced, widowed, separated | 23 (0.4) |  | 75 (0.5) |  |
|  | Single | 225 (3.4) |  | 776 (5.5) |  |
|  | Missing | 19 (0.3) |  | 64 (0.5) |  |
| Education, n (%) | |  |  |  | <0.001 |
|  | Lower secondary (≤9 years) | 246 (3.7) |  | 648 (4.6) |  |
|  | Upper secondary (10-12 years) | 2,755 (41.3) |  | 5,757 (40.9) |  |
|  | Tertiary (>12 years) | 3,534 (52.9) |  | 6,447 (45.8) |  |
|  | Missing | 144 (2.2) |  | 1,222 (8.7) |  |
| Childhood adversities, n (%)* | |  |  |  |  |
|  | Perceiving childhood as difficult | 341 (5.1) |  | 822 (6.4) | <0.001 |
|  | Parental divorce | 1,251 (18.8) |  | 1,976 (15.3) | <0.001 |
|  | Parental death | 331 (5.0) |  | 776 (6.0) | <0.01 |
|  | Dysfunctional family environment | 768 (13.8) |  | 1,629 (15.0) | <0.04 |
|  | Struggle with bad memories | 274 (4.9) |  | 650 (6.0) | <0.01 |
|  | Lack of trusted adult | 840 (15.1) |  | 2,037 (18,8) | <0.001 |
| * Percentage among HUNT participants without missing exposure.  **Information on birthyear, maternal age, parity, marital status and employment status at a woman’s last birth was retrieved from The Medical Birth Registry of Norway (MBRN). Information on education from HUNT surveys.  ***Differences between included and excluded women were assessed with a two sided t-test for continuous variables and chi-square test for categorical variables. | | | | | |
