# Supplementary material for: Adverse childhood experiences and pre-pregnancy body mass index in the HUNT study: A population-based cohort study
Source: PLoS One. 2023 May 2;18(5):e0285160. doi: 10.1371/journal.pone.0285160 (PMC10153725; doi:10.1371/journal.pone.0285160)
Supplement: S2 Table — (DOCX) [file pone.0285160.s004.docx]

| **Table S2. Crude odds ratios and 95% confidence intervals of pre-pregnancy BMI according to adverse childhood experiences** | | | | | | |
| --- | --- | --- | --- | --- | --- | --- |
| **Adverse childhood experiences** | | | n (total) | n (exposed) | OR* | 95% CI |
| **Perceiving childhood as difficult** | | | 6,679 | 341 |  |  |
|  | Normal weight (BMI 18.5-24.9) | | 4,223 | 195 | 1.00 | - |
|  | Underweight (BMI <18.5) | | 154 | 13 | 1.90 | 1.06-3.42 |
|  | Overweight (BMI 25-29.9) | | 1,577 | 82 | 1.13 | 0.87-1.48 |
|  | Obese (BMI ≥30) | | 725 | 51 | 1.56 | 1.14-2.15 |
|  |  | Obesity class 1 (BMI 30-34.9) | 518 | 28 | 1.18 | 0.79-1.77 |
|  |  | Obesity class 2 (BMI 35-39.9) | 164 | 16 | 2.23 | 1.31-3.81 |
|  |  | Obesity class 3 (BMI ≥40) | 43 | 7 | 4.02 | 1.76-9.14 |
| **Parental divorce during childhood** | | | 6,661 | 1,251 |  |  |
|  | Normal weight (BMI 18.5-24.9) | | 4,214 | 708 | 1.00 | - |
|  | Underweight (BMI <18.5) | | 153 | 34 | 1.41 | 0.96-2.09 |
|  | Overweight (BMI 25-29.9) | | 1,567 | 321 | 1.28 | 1.10-1.48 |
|  | Obese (BMI ≥30) | | 723 | 188 | 1.74 | 1.45-2.09 |
|  |  | Obesity class 1 (BMI 30-34.9) | 517 | 124 | 1.56 | 1.26-1.94 |
|  |  | Obesity class 2 (BMI 35-39.9) | 164 | 47 | 1.99 | 1.40-2.82 |
|  |  | Obesity class 3 (BMI ≥40) | 42 | 17 | 3.37 | 1.80-6.27 |
| **Parental death during childhood** | | | 6,664 | 331 |  |  |
|  | Normal weight ((BMI 18.5-24.9) | | 4,212 | 216 | 1,00 | - |
|  | Underweight (BMI <18.5) | | 154 | 6 | 0.75 | 0.33-1.72 |
|  | Overweight (BMI 25-29.9) | | 1,574 | 74 | 0.91 | 0.70-1.20 |
|  | Obese (BMI ≥30) | | 724 | 35 | 1.94 | 0.65-1.36 |
|  |  | Obesity class 1 (BMI 30-34.9) | 517 | 26 | 0.98 | 0.65-1.49 |
|  |  | Obesity class 2 (BMI 35-39.9) | 164 | 8 | 0.95 | 0.46-1.96 |
|  |  | Obesity class 3 (BMI ≥40) | 43 | 1 | 0.44 | 0.06-3.22 |
| **Dysfunctional family environment** | | | 5,575 | 768 |  |  |
|  | Normal weight ((BMI 18.5-24.9) | | 3,557 | 476 | 1.00 | - |
|  | Underweight (BMI <18.5) | | 125 | 21 | 1.31 | 0.81-2.11 |
|  | Overweight (BMI 25-29.9) | | 1.302 | 182 | 1.05 | 0.88-1.26 |
|  | Obese (BMI ≥30) | | 591 | 89 | 1.15 | 0.90-1.47 |
|  |  | Obesity class 1 (BMI 30-34.9) | 425 | 52 | 0.90 | 0.66-1.22 |
|  |  | Obesity class 2 (BMI 35-39.9) | 135 | 29 | 1.77 | 1.16-2.70 |
|  |  | Obesity class 3 (BMI ≥40) | 31 | 8 | 2.25 | 1.00-5.06 |
| **Struggle with bad memories** | | | 5,571 | 274 |  |  |
|  | Normal weight ((BMI 18.5-24.9) | | 3,558 | 148 | 1.00 | - |
|  | Underweight (BMI <18.5) | | 125 | 8 | 1.58 | 0.0.76-3.29 |
|  | Overweight (BMI 25-29.9) | | 1,302 | 76 | 1.43 | 1.07-1.90 |
|  | Obese (BMI ≥30) | | 586 | 42 | 1.78 | 1.25-2.54 |
|  |  | Obesity class 1 (BMI 30-34.9) | 421 | 26 | 1.52 | 0.99-2.33 |
|  |  | Obesity class 2 (BMI 35-39.9) | 135 | 13 | 2.46 | 1.35-4.45 |
|  |  | Obesity class 3 (BMI ≥40) | 30 | 3 | 2.56 | 0.77-8.53 |
| **Lack of trusted** **adult during childhood** | | | 5,552 | 840 |  |  |
|  | Normal weight ((BMI 18.5-24.9) | | 3,544 | 526 | 1.00 | - |
|  | Underweight (BMI <18.5) | | 124 | 21 | 1.17 | 0.73-1.89 |
|  | Overweight (BMI 25-29.9) | | 1.300 | 196 | 1.02 | 0.85-1.22 |
|  | Obese (BMI ≥30) | | 584 | 97 | 1.14 | 0.90-1.45 |
|  |  | Obesity class 1 (BMI 30-34.9) | 420 | 60 | 0.96 | 0.72-1.28 |
|  |  | Obesity class 2 (BMI 35-39.9) | 134 | 29 | 1.58 | 1.04-2.42 |
|  |  | Obesity class 3 (BMI ≥40) | 30 | 8 | 2.09 | 0.92-4.71 |
| BMI, body mass index; CI, confidence interval; OR, odds ratio. | | | | | | |
