# Supplementary material for: Adverse childhood experiences and pre-pregnancy body mass index in the HUNT study: A population-based cohort study
Source: PLoS One. 2023 May 2;18(5):e0285160. doi: 10.1371/journal.pone.0285160 (PMC10153725; doi:10.1371/journal.pone.0285160)
Supplement: S3 Table — (DOCX) [file pone.0285160.s005.docx]

| **Table S3.** Associations of maternal childhood experiences with pre-pregnancy BMI in a subpopulation of younger women born after 1980 | | | | | | |
| --- | --- | --- | --- | --- | --- | --- |
| **Adverse childhood experiences** | | | n (total) | n (exposed) | OR* | 95% CI |
| **Perceiving childhood as difficult** | | | 2,980 | 158 |  |  |
|  | Normal weight (BMI 18.5-24.9) | | 1,695 | 79 | 1.00 | - |
|  | Underweight (BMI <18.5) | | 67 | 6 | 1.86 | 0.78-4.46 |
|  | Overweight (BMI 25-29.9) | | 741 | 42 | 1.23 | 0.84-1.81 |
|  | Obese (BMI ≥30) | | 405 | 31 | 1.71 | 1.11-2.63 |
|  |  | Obesity class 1 (BMI 30-34.9) | 291 | 19 | 1.45 | 0.86-2.43 |
|  |  | Obesity class 2 (BMI 35-39.9) | 93 | 10 | 2.45 | 1.22-4.93 |
|  |  | Obesity class 3 (BMI ≥40) | 21 | 2 | 2.31 | 0.53-10.2 |
| **Parental divorce during childhood** | | |  |  |  |  |
|  | Normal weight (BMI 18.5-24.9) | | 1.692 | 466 | 1.00 | - |
|  | Underweight (BMI <18.5) | | 67 | 26 | 1.46 | 0.87-2.44 |
|  | Overweight (BMI 25-29.9) | | 737 | 220 | 1.14 | 0.94-1.38 |
|  | Obese (BMI ≥30) | | 404 | 142 | 1.45 | 1.15-1.83 |
|  |  | Obesity class 1 (BMI 30-34.9) | 290 | 98 | 1.39 | 1.06-1.81 |
|  |  | Obesity class 2 (BMI 35-39.9) | 93 | 34 | 1.45 | 0.93-2.26 |
|  |  | Obesity class 3 (BMI ≥40) | 21 | 10 | 2.61 | 1.09-6.27 |
| **Parental death during childhood** | | | 2,903 | 108 |  |  |
|  | Normal weight ((BMI 18.5-24.9) | | 1,692 | 67 | 1,00 | - |
|  | Underweight (BMI <18.5) | | 67 | 3 | 1.26 | 0.38-4.16 |
|  | Overweight (BMI 25-29.9) | | 740 | 21 | 0.72 | 0.44-1.18 |
|  | Obese (BMI ≥30) | | 404 | 17 | 1.07 | 0.62-1.84 |
|  |  | Obesity class 1 (BMI 30-34.9) | 290 | 12 | 1.04 | 0.56-1.96 |
|  |  | Obesity class 2 (BMI 35-39.9) | 93 | 4 | 1.09 | 0.39-3.07 |
|  |  | Obesity class 3 (BMI ≥40) | 21 | 1 | 1.15 | 0.15-8.76 |
| **Dysfunctional family environment** | | | 2,370 | 371 |  |  |
|  | Normal weight ((BMI 18.5-24.9) | | 1,369 | 203 | 1.00 | - |
|  | Underweight (BMI <18.5) | | 56 | 14 | 1.84 | 0.98-3.44 |
|  | Overweight (BMI 25-29.9) | | 614 | 96 | 1.07 | 0.82-1.39 |
|  | Obese (BMI ≥30) | | 331 | 59 | 1.22 | 0.89-1.68 |
|  |  | Obesity class 1 (BMI 30-34.9) | 237 | 35 | 0.82 | 0.60-1.11 |
|  |  | Obesity class 2 (BMI 35-39.9) | 78 | 19 | 1.84 | 1.07-3.15 |
|  |  | Obesity class 3 (BMI ≥40) | 16 | 4 | 1.87 | 0.60-5.87 |
| **Struggle with bad memories** | | | 2,368 | 150 |  |  |
|  | Normal weight ((BMI 18.5-24.9) | | 1,369 | 74 | 1.00 | - |
|  | Underweight (BMI <18.5) | | 56 | 5 | 1.61 | 0.62-4.19 |
|  | Overweight (BMI 25-29.9) | | 613 | 46 | 1.50 | 1.02-2.21 |
|  | Obese (BMI ≥30) | | 330 | 28 | 1.71 | 1.08-2.70 |
|  |  | Obesity class 1 (BMI 30-34.9) | 236 | 19 | 1.38 | 0.89-2.14 |
|  |  | Obesity class 2 (BMI 35-39.9) | 78 | 8 | 2.05 | 0.94-4.44 |
|  |  | Obesity class 3 (BMI ≥40) | 16 | 1 | 1.30 | 0.17-10.02 |
| **Lack of trusted** **adult during childhood** | | | 2,361 | 317 |  |  |
|  | Normal weight ((BMI 18.5-24.9) | | 1,365 | 176 | 1.00 | - |
|  | Underweight (BMI <18.5) | | 56 | 13 | 2.04 | 1.07-3.89 |
|  | Overweight (BMI 25-29.9) | | 612 | 79 | 0.99 | 0.75-1.32 |
|  | Obese (BMI ≥30) | | 328 | 49 | 1.17 | 0.83-1.66 |
|  |  | Obesity class 1 (BMI 30-34.9) | 235 | 31 | 1.01 | 0.67-1.52 |
|  |  | Obesity class 2 (BMI 35-39.9) | 78 | 15 | 1.63 | 0.91-2.93 |
|  |  | Obesity class 3 (BMI ≥40) | 15 | 3 | 1.60 | 0.44-5.76 |
| BMI, body mass index; CI, confidence interval; OR, odds ratio.  *Models are adjusted for age and birthyear. | | | | | | |
