# Supplementary material for: Adverse childhood experiences and pre-pregnancy body mass index in the HUNT study: A population-based cohort study
Source: PLoS One. 2023 May 2;18(5):e0285160. doi: 10.1371/journal.pone.0285160 (PMC10153725; doi:10.1371/journal.pone.0285160)
Supplement: S4 Table — (DOCX) [file pone.0285160.s006.docx]

| **S4 Table. Associations of perceiving childhood as difficult with pre-pregnancy BMI in a subpopulation of women with repeated information from HUNT3 and HUNT4** | | | | | | |
| --- | --- | --- | --- | --- | --- | --- |
| **Adverse childhood experiences** | | | n (total) | n (exposed) | OR* | 95% CI |
| **Perceiving childhood as difficult (based on first reported answer in HUNT3)** | | | | | | |
|  | | | 6,679 | 341 |  |  |
|  | Normal weight (BMI 18.5-24.9) | | 4,223 | 195 | 1.00 | - |
|  | Underweight (BMI <18.5) | | 154 | 13 | 1.78 | 0.99-3.22 |
|  | Overweight (BMI 25-29.9) | | 1,577 | 82 | 1.15 | 0.88-1.50 |
|  | Obese (BMI ≥30) | | 725 | 51 | 1.58 | 1.14-2.20 |
|  |  | Obesity class 1 (BMI 30-34.9) | 518 | 28 | 1.19 | 0.79-1.81 |
|  |  | Obesity class 2 (BMI 35-39.9) | 164 | 16 | 2.32 | 1.35-4.01 |
|  |  | Obesity class 3 (BMI ≥40) | 43 | 7 | 4.62 | 2.0-10.65 |
|  |  |  |  |  |  |  |
| **Perceiving childhood as difficult (ever reported perceiving childhood as difficult in HUNT3 or HUNT4)** | | | | | | |
|  | | | 6,679 | 384 |  |  |
|  | Normal weight (BMI 18.5-24.9) | | 4,223 | 223 | 1.00 | - |
|  | Underweight (BMI <18.5) | | 154 | 14 | 1.69 | 0.96-2.98 |
|  | Overweight (BMI 25-29.9) | | 1,577 | 93 | 1.16 | 0.91-1.49 |
|  | Obese (BMI ≥30) | | 725 | 53 | 1.48 | 1.08-2.04 |
|  |  | Obesity class 1 (BMI 30-34.9) | 518 | 30 | 1.15 | 0.77-1.72 |
|  |  | Obesity class 2 (BMI 35-39.9) | 164 | 16 | 2.10 | 1.22-3.60 |
|  |  | Obesity class 3 (BMI ≥40) | 43 | 7 | 4.10 | 1.78-9.42 |
|  |  |  |  |  |  |  |
| BMI, body mass index; CI, confidence interval; OR, odds ratio.  *Models are adjusted for age and birthyear. | | | | | | |
