# Supplementary material for: Adverse childhood experiences and pre-pregnancy body mass index in the HUNT study: A population-based cohort study
Source: PLoS One. 2023 May 2;18(5):e0285160. doi: 10.1371/journal.pone.0285160 (PMC10153725; doi:10.1371/journal.pone.0285160)
Supplement: S5 Table — (DOCX) [file pone.0285160.s007.docx]

| **S5 Table. Categorization of pre-pregnancy BMI category based on BMI reported to the MBRN and BMI measured at HUNT (n=326)** | | | | |
| --- | --- | --- | --- | --- |
| **BMI from HUNT** | **BMI from MBRN** | | | |
|  | Underweight | Normal weight | Overweight | Obese |
| Underweight (n=9) | 77.9% | 22.2% | 0% | 0% |
| Normal weight (n=185) | 1.1% | 91.9% | 7.0% | 0% |
| Overweight (n=84) | 0% | 21.4% | 72.6% | 6.0% |
| Obese (n=48) | 0% | 2.1% | 14.6% | 83.3% |
| *Agreement proportion of women in each pre-pregnancy BMI category who were truly underweight, truly normal weight, truly overweight, truly obese when BMI information from MBRN was compared to objective measured BMI from a HUNT examination.  Abbreviations: BMI, body mass index; MBRN, Medical Birth Registry of Norway.  Pre-pregnancy BMI categories were underweight (< 18.5 kg/m^2^), normal weight (18.5 to < 25 kg/m^2^), overweight (25 to < 30 kg/m^2^) and obesity (≥ 30 kg/m^2^) based on WHOs classification of nutritional status. | | | | |
